# Regulation of metabolism by sirtuins

**DOI:** 10.1186/1753-6561-6-S3-O9

**Published:** 2012-06-01

**Authors:** Marcia Haigis

**Affiliations:** 1 Department of Cell Biology, Harvard Medical School, Boston, MA 02115, USA

## 

Mitochondrial sirtuins are NAD-dependent enzymes that bind and regulate numerous metabolic pathways within the mitochondria. For example, SIRT3 functions as an NAD-dependent deacetylase that binds and activates numerous oxidative pathways. We have discovered that sirtuins regulate metabolic pathways important in tumor cell metabolism. One hallmark feature of tumor cells is a shift from oxidative to glycolytic metabolism, and this reliance on aerobic glycolysis to support cell growth is known as the Warburg effect. We have discovered that SIRT3 has an additional effect on cellular metabolism by repressing cellular glycolysis through the regulation of HIF1α, a transcription factor that increases gene expression of glycolytic targets. SIRT3 null cells exhibit metabolic and genetic features of the Warburg effect and enhanced tumorigenecity *in vivo.* Likewise, SIRT3 overexpression reduces glycolysis in tumor cells. In sum, a better understanding of sirtuin-mediated regulation may identify novel ways to therapeutically target tumor metabolism.

